# Very preterm gut microbiota development from the first week of life to 3.5 years of age: a prospective longitudinal multicenter study

**DOI:** 10.1128/spectrum.01636-24

**Published:** 2025-02-19

**Authors:** Gaël Toubon, Constance Patin, Johanne Delannoy, Jean-Christophe Rozé, Frédéric Barbut, Pierre-Yves Ancel, Marie-Aline Charles, Marie-José Butel, Patricia Lepage, Julio Aires

**Affiliations:** 1 INSERM, UMR1153 Centre de Recherche Épidémiologie et StatistiqueS (CRESS), Université Paris Cité, Paris, France; 2 INSERM, UMR-S 1139, Physiopathologie et Pharmacotoxicologie Placentaire Humaine Microbiote Pré & Postnatal, Université Paris Cité,, Paris, France; 3 FHU PREMA, “Fighting Prematurity”, Paris, France; 4 INRAE, UMR 1319, AgrosParisTech, Institut Micalis, Université Paris-Saclay, Paris, France; 5 INRAE, UMR 1280, Physiologie des Adaptations Nutritionnelles (PhAN), Université Hospitalière de Nantes, Nantes, France; Korea University, Seoul, South Korea

**Keywords:** very preterm neonates, gut microbiota, enterotypes, longitudinal study, 16S rRNA gene sequencing, gestational age, delivery mode, birth weight, practice of skin-to-skin contact, antibiotherapy, maternal BMI

## Abstract

This study analyzed the longitudinal evolution of intestinal microbiota in very preterm neonates (PN) during and after their hospitalization. The bacterial 16S rRNA gene sequencing approach was applied for the analysis of fecal samples (*n* = 1,307) from 596 PN. Samples were collected at one week after birth, at one month, at the neonatal intensive care unit discharge, and at 3.5 years of age. Over time, the intestinal microbiota of the infants matured progressively, with increasing alpha diversity and decreasing beta diversity. Based on a Dirichlet multinomial mixture clustering approach (DMM), during hospitalization, infants progressed among ten different clusters. At 3.5 years of age, only three clusters were identified. The influence of the gestational age, the neonatal antibiotic administration, and the maternal antibiotic therapy during delivery on the gut microbiota varied over time and depended on the sampling period. Preconceptional maternal body mass index (BMI) was associated with the gut microbiota of infants during the hospitalization period and at 3.5 years of age. Infants with a lower gestational age or those born by Cesarean section shifted between clusters more frequently. Using PICRUSt2, the inferred metabolic pathways revealed a change in the functional capacities of the intestinal microbiota over time. We found that preconceptional maternal BMI was the only consistent perinatal factor influencing the development of the gut microbiota over time. After hospital discharge, infants exhibited a transition toward a microbiota community similar to that of adults by 3.5 years of age, in accordance with the functional metabolic pathways of the gut microbiota.

**IMPORTANCE:**

This study is among the very few reports analyzing the gut microbiota development in very preterm infants over time in a large, multicenter population of 596 children from a well-described nationwide birth cohort, with a follow-up until the age of 3.5 years. The maturation of the intestinal microbiota was confirmed to occur over time, with increased alpha diversity and decreased beta diversity. Specifically, 13 microbiota clusters were identified during the hospitalization period, while and only three clusters were observed at 3.5 years. Infants born prematurely or via Cesarean section exhibited a less stable microbiota, frequently shifting clusters. A number of perinatal factors were identified as influencing the development of the microbiota. Among these, the preconceptional maternal BMI emerged as the only consistent factor up to 3.5 years. The metabolic pathways of the microbiota evolved over time, in accordance with the maturation of the gut microbiota.

## INTRODUCTION

The dynamics of the gut microbiota during early development can have a significant impact on children’s health as they grow up (1, 2). In infants, the microbial community displays a less complex but more variable pattern than in adults, where the gut microbiota is considered relatively stable and resilient in the absence of stressors (3). The maturation of the gut microbiota in full-term infants is a longitudinal process that results in the formation of age-specific community types. This process occurs over time and is characterized by three distinct phases: a developmental phase (up to 14 months), a transitional phase (between 15 and 30 months), and a stabilization phase (up to 46 months) (4). It is acknowledged that an adult-like microbiota stabilization occurs around the age of 5 years (5). A number of studies have reported on the factors that influence the establishment of gut microbiota in early life (1, 6–8). These include prolonged hospitalization, diet, antibiotic exposure, and mode of delivery (9–13). In preterm (less than 37 weeks of gestational age) and very preterm (less than 32 weeks of gestational age) neonates, the development of the gut microbiota differs from that in full-term neonates due to a number of factors, including birth conditions and extended exposure to the neonatal intensive care unit (NICU) environment and practices (14, 15). The establishment of the gut microbiota in preterm infants is characterized by a dysbiotic state compared to that of full-term infants. This is characterized by lower alpha diversity; an overrepresentation of pathobionts such as *Enterobacter, Enterococcus*, and *Staphylococcus*; and a delay in the colonization of commensal bacteria, particularly *Bifidobacterium* and *Bacteroides* (7, 16–19). Previously, we and others have shown that very preterm neonates can be stratified into distinct enterotypes (or clusters) driven by specific genera, including *Enterobacter*, *Clostridium sensu stricto 1*, *Escherichia-Shigella*, *Enterococcus*, and *Staphylococcus*, based on their gut microbiota (20–22). By approximately 3 years of age, the gut microbiota becomes more stable and is characterized by only two discrete enterotypes, one dominated by *Bacteroides* and the other by *Prevotella* (8, 22).

A limited number of studies have examined the longitudinal patterns and maturation of the very preterm gut microbiota. There is a lack of data on this topic following the period of hospitalization, when preterm infants grow in their family environment. The present study provides data on the evolution of the gut microbiota up to 3.5 years of age in a large French multicenter cohort of very preterm infants. A total of 596 very preterm neonates (PN) were included in this study, with fecal samples collected at 1 week, 1 month of life, upon NICU discharge, and at 3.5 years of age. The impact of perinatal factors, including gestational age, mode of delivery, birth weight, maternal milk intake, practice of skin-to-skin contact, neonatal and maternal antibiotic therapy, and maternal body mass index (BMI), on the development of the gut microbiota was investigated in a cross-sectional and longitudinal manner. Furthermore, the functional prediction content based on the 16S rRNA sequencing data set was employed to assess and compare the inferred functional capabilities of the gut microbiota over time.

## MATERIALS AND METHODS

### Study

The EPIFLORE study included 24 voluntary NICUs from the EPIPAGE 2 French national birth cohort that enrolled very preterm neonates (< 32 weeks of gestation) in 2011 (20, 23, 24). For the purposes of this study, eligible infants (*n* = 596) were those alive at week 4 after birth, hospitalized in one of the 24 voluntary NICUs, and who provided at least one fecal sample with positive DNA amplification during the polymerase chain reaction (PCR) procedure targeting the bacterial 16S rRNA gene. Stool samples (*n* = 1,307) were collected at four time points: the first week of life (1W), 1 month (1M), at NICU discharge (D), and at 3.5 years of age (3.5Y). The EPIFLORE study was granted approval from the National Data Protection Authority (Commission Nationale de l’Informatique et des Libertés [CNIL]), by the national advisory committee on information processing in health research (Comité Consultatif sur le Traitement de l’Information en matière de Recherche dans le domaine de la Santé [CCTIRS]), and by the Committee for the Protection of People Participating in Biomedical Research (Comité de Protection des Personnes [CPP]). The recruitment and data collection procedures were initiated only after the families had received comprehensive information about the study and agreed to participate in the cohort with informed oral consent.

### DNA extraction, sequencing, and data processing

The stool samples were collected and stored at −80°C until total fecal DNA extraction was performed as recommended by the International Human Microbiome Standards operating procedure (8). Sequencing was performed as previously described (8). Raw sequences were analyzed using the pipeline FROGS version 3.2 pipeline from the Galaxy software framework (25). The sequences were filtered for length (minimum length = 380 bp; maximum length = 500 bp) after trimming barcodes and removing chimeras. Subsequently, the swarm clustering method, implemented in the FROGS version 3.2 pipeline, was used to cluster the sequences into operational taxonomic units (OTUs). OTUs with a relative abundance of less than 0.005% of all the sequences were discarded (26). Finally, the Silva 138.1 pintail100 database was used to assign taxonomic affiliation to OTUs. A total of 27,440,655 reads were obtained from 16S rRNA gene sequencing, with a median of 23,339 reads per sample, distributed across 451 OTUs. To account for the uneven sampling depth, each sample was rarefied to a depth of 4,890 reads, resulting in the exclusion of five fecal samples. All subsequent analyses were conducted on the rarefied data, unless otherwise specified.

### Microbiota profiling and functional metabolic pathway prediction

A differential abundance testing analysis was performed to evaluate significant changes in composition at the genus level between the different fecal sampling points. To account for potential heterogeneity in the results (27, 28), two different methods were utilized: the Analysis of Compositions of Microbiomes with Bias Correction (ANCOM-BC) (29) and the ANOVA-Like Differential Expression tool for compositional data (ALDEx2) (30). For both methods, differences in abundance were based on nonrarefied counts as each method used its own normalization. Furthermore, only genera with an abundance of 0.1% in at least 1% of the samples and consistently identified by both methods were considered. The richness and alpha-diversity of the gut microbiota were evaluated using the Chao1 and Shannon indexes, respectively. Beta-diversity was evaluated by calculating dissimilarity matrices using Bray–Curtis and UniFrac distances. PICRUSt2 software was used to infer functions based on the 16S rRNA gene sequencing data set (31), enabling the generation of normalized Kyoto Encyclopedia of Genes and Genomes (KEGG) pathways, according to known/predicted 16S rRNA gene copy numbers. The inferred functional profiles of microbial communities at the third hierarchy level (KEGG pathways) were analyzed using Statistical Analysis of Metagenomic Profiles (STAMP) v2.1.3 (32).

### Statistical analysis

To assess the cross-sectional associations between perinatal factors and alpha-diversity metrics, Wilcoxon rank-sum tests and Kruskal–Wallis tests were employed, followed by Dunn’s test for multiple comparisons. All longitudinal analyses were conducted with at least two sampling time points. For longitudinal associations, multivariable mixed linear regression models were employed, with infant identification serving as a random intercept to account for repeated measures. The models were adjusted for maternal age, level of education, and the mother’s country of birth. Permutational multivariate analysis of variance (PERMANOVA) was used to test the contribution of perinatal factors to the variation in the gut microbiota composition. The interindividual variability of the gut microbiota based on taxonomic profiling at different sampling time points was visualized using nonmetric multidimensional scaling (NMDS), based on the Bray–Curtis dissimilarity matrix and tested using the PERMANOVA test. The interindividual variability of the gut microbiota based on functional profiling at different sampling time points was visualized using principal component analysis (PCA) and tested using the multivariate analysis of variance (MANOVA) test. The progression of the microbial community was determined by clustering rarefied counts using the Dirichlet multinomial mixture model (DMM) (33). The number of clusters was determined based on the lowest Laplace approximation score. Subsequently, samples were assigned to a specific community type based on their maximum posterior probability. Furthermore, partitioned data (1W/1M; 1M/D) were used to calculate the transition between clusters for each subject. This provided summary measures of microbiome stability over time. The distribution between clusters in categorical factors was assessed using Fisher’s exact test. The inferences drawn from the functional pathway differences across sampling points were evaluated using Welch’s test. In order to identify the most significantly affected features, a q-value of <0.05 was employed in conjunction with an effect size filter (difference between proportions effect size <0.2). Benjamini–Hochberg’s FDR (false discovery rate) method was used to correct for multiple testing in Dunn’s test, PERMANOVA, and Welch’s tests. A statistical association of *P* < 0.05 was set for all statistical analyses. All statistical analyses were performed using the R environment version 4.0.4 (R Foundation), except for the differential abundance testing for functional pathways that was performed using STAMP v2.1.3.

## RESULTS

### Subject characteristics

The characteristics of the 596 very preterm neonates included in the present study are presented in Table 1. A total of 1,037 stool samples were analyzed, included 137 samples obtained at 1W (median age of 7 days [d] [interquartile range [IQR], 6–9 d]), 480 samples obtained at 1M (median age of 24 d [IQR, 22–27 d]), 212 samples obtained at D (median age of 53 d [IQR, 39–70.25 d]), and 208 samples obtained at 3.5Y (median age of 1,297 d [IQR, 1,284–1,318 d]).

**TABLE 1:** Descriptive data of the EPIFLORE population^*a*^^,^^*b*^

Total cohort	(*N* = 596)			
Maternal age (years)				
<25	97 (16.3%)			
[25–35]	341 (57.2%)			
≥35	136 (22.8%)			
Missing	22 (3.7%)			
Maternal level of education				
< High school	157 (26.3%)			
High school	115 (19.3%)			
High school diploma +1 + 2	119 (20.0%)			
> High school diploma +3	168 (28.2%)			
Missing	37 (6.2%)			
Mother born in France				
Yes	442 (74.2%)			
No	151 (25.3%)			
Missing	3 (0.5%)			
Maternal BMI				
Underweight	39 (6.5%)			
Normal	332 (55.7%)			
Overweight	110 (18.5%)			
Obese	73 (12.2%)			
Missing	42 (7.0%)			
Gestational age (weeks)				
24–26	63 (10.6%)			
27–29	220 (36.9%)			
30–32	313 (52.5%)			
Delivery mode				
Vaginal	208 (34.9%)			
C-section	385 (64.6%)			
Missing	3 (0.5%)			
Birth weight (g)				
Median (IQR)	1,122.5 (903–1,408)			
Sex				
Boys	321 (53.9%)			
Girls	275 (46.1%)			
Neonatal primary antibiotherapy^*c*^				
No	123 (20.6%)			
Yes	408 (68.5%)			
Missing	65 (10.9%)			
Mother intra-partum antibiotherapy				
No	325 (54.5%)			
Yes	195 (32.7%)			
Missing	76 (12.8%)			
Mother antenatal antibiotherapy				
No	291 (48.8%)			
Yes	299 (50.2%)			
Missing	6 (1.0%)			
Practice of skin-to-skin contact				
Yes	335 (56.2%)			
No	229 (38.4%)			
Missing	32 (5.4%)			
NEC				
No	576 (96.6%)			
Yes	19 (3.2%)			
Missing	1 (0.2%)			
LOS				
No	449 (75.3%)			
Yes	140 (23.5%)			
Missing	7 (1.2%)			
Intake of breastmilk	1W	1M	D	3.5Y
No	6 (4.4%)	44 (9.2%)	64 (30.2%)	160 (76.9%)
Yes	85 (62.0%)	256 (53.3%)	74 (34.9%)	0 (0%)
Missing	46 (33.6%)	180 (37.5%)	74 (34.9%)	48 (23.1%)
Age at fecal sampling (days)				
Mean (SD)	7.55 (2.58)	24.8 (5.05)	58.4 (28.9)	1300 (31.0)
Missing	0 (0%)	2 (0.4%)	0 (0%)	12 (5.8%)

^*a*^ The data presented are number of events (percentages) for categorical variables and the median with interquartile range (IQR) or mean (SD) for continuous variables. Missing values are noted, if any. The variable “Intake of breastmilk” refers to any human milk intake, regardless of the source (own mother or donor), mode (bottle or breast), and whether it is exclusive or not at the time of sampling.

^*b*^ NEC: necrotizing enterocolitis. LOS: late-onset sepsis. BMI: body mass index based on WHO standard cut-offs (34). 1W: stool sample collected during the first week of life. 1M: stool sample collected at 1 month of life. D: stool sample collected at NICU discharge. 3.5Y: stool sample collected at 3.5 years of age.

^*c*^ Neonatal primary antibiotherapy corresponds to the antibiotics received during the first 3 days of life.

### Gut microbiota characteristics

**Fig 1:**
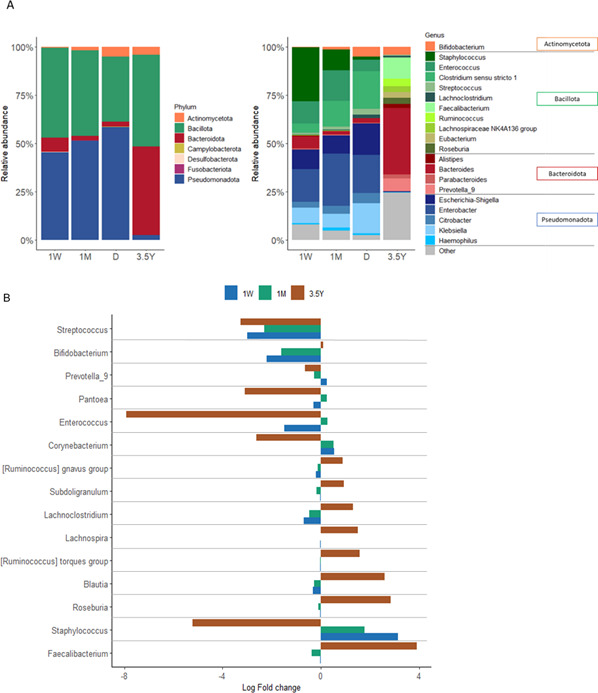
Gut microbiota composition in very preterm infants from 1 week of life to 3.5 years of age. (**A**) Relative abundance of taxa at the phylum level (left) and the 20 most abundant genera (right) according to the sampling time points 1 week (1W; *n* = 137), 1 month (1M; *n* = 480), NICU discharge (D; *n* = 212), and 3.5 years of age (3.5Y; *n* = 208). (**B**) Bar plots of significant ANCOM-BC log-transformed in abundance results at the genus level for each sampling time point and compared to the D sample chosen as the reference. Bar plots are colored based on the sampling time points, and the direction of the bars indicates positive or negative log fold-change in abundance. Only genera that were found differentially abundant according to both ANCOM-BC and ALDEx2 methods are presented. The conclusions regarding higher or lower ALDEx2 abundance were consistent with ANCOM-BC results. ANCOM-BC and ALDEx2 data are presented in Table S1.

During the period of hospitalization (1W, 1M, and D samples), the gut microbiota was dominated by two major phyla, Pseudomonadota and Bacillota (Fig. 1A). At the 1W, 1M, and D sampling times, the relative abundance of Pseudomonadota was 45%, 51%, and 59%, respectively, while that of Bacillota was 46%, 44%, and 34%. At 3.5Y, Bacillota (47%) and Bacteroidota (46%) were the predominant phyla in the gut microbiota. At the genus level, there was a marked decline in the proportion of *Staphylococcus* from 1W to D, from 28% to less than 1% (Fig. 1A). In contrast, the proportion of *Clostridium sensu stricto* 1 increased from 1W to D (increasing from 5% to 20%). Within the Pseudomonadota, the predominant genus detected at 1W and 1M was *Enterobacter*. However, its proportions slightly decreased during the period of hospitalization, as well as for *Escherichia-Shigella*, while *Klebsiella* proportions increased. During the hospitalization, both *Bacteroides* and *Bifidobacterium* relative abundances remained at low levels (< 7%). At 3.5Y, there was a lower proportion of facultative anaerobic bacteria and higher levels of anaerobic bacteria, with the majority belonging to the genera *Faecalibacterium* and *Bacteroides*. The data from both ANCOM-BC and ALDEx2 indicate that 15 concordant, overlapping abundant genera were significantly different for each sampling point when compared to D, which served as a reference (Fig. 1B). Samples collected at D were considered the reference point as they represent the intermediate time point between the earliest period of hospitalization and childhood at 3.5 years. A significant log-fold change in the abundance of *Staphylococcus* was observed, with a decrease from 3.1 at 1W to 1.8 at 1M (*P* < 0.0001), in comparison to the D samples (Fig. 1B; Table S1). This is consistent with the relative abundance of taxa shown in Fig. 1A. Notable fluctuations in abundances were observed during the hospitalization period, including an increase in *Streptococcus, Bifidobacterium,* and *Lachnoclostridium* and a decrease in *Corynebacterium* (Fig. 1B). At 3.5Y, the most significant changes in abundance were increases in *Faecalibacterium, Ruminococcus, Blautia*, and *Roseburia* (all *P* < 0.0001) and a notable decrease in *Enteroccocus* (*P* < 0.0001) (Fig. 1B; Table S1).

**Fig 2:**
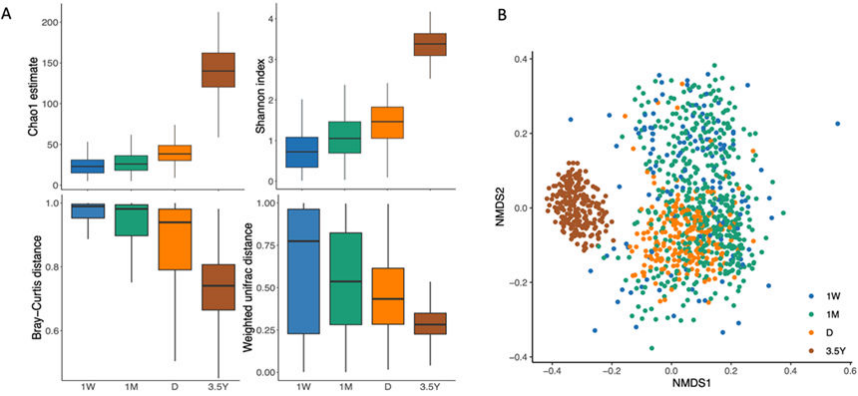
Diversity development of the very preterm infants gut microbiota from 1 week of life to 3.5 years of age. (**A**) Box plots of alpha-diversity (top) measured as Chao1 and Shannon indexes and beta-diversity (bottom) assessed by Bray–Curtis and Weighted UniFrac pairwise distances for fecal samples at 1 week (1W; *n* = 137), 1 month (1M; *n* = 480), NICU discharge (D; *n* = 212), and 3.5 years of age (3.5Y; *n* = 208). All groups are significantly different from each other (*P* < 0.05) (see Table S2). Box plots show minimum and maximum values, 25% and 75% quartiles, and the median. (**B**) First and second principal coordinates of nonmetric multidimensional scaling (NMDS) dimension reduction for Bray–Curtis distance (stress value = 0.08). Each point indicates an infant fecal sample, colored by the time of sampling.

The alpha diversity of the gut microbiota increased significantly over time between the different sampling time points (*P* < 0.001, both indexes) (Fig. 2A; Table S2A). Beta-diversity analysis showed that the composition of the gut microbiota underwent a significant change over time (*P* = 0.001, both distances) (Fig. 2; Table S2B). As the infant grew older, the samples within the age group became more similar, as evidenced by the reduction in pairwise dissimilarity distances (Fig. 2A). Despite the existence of intervariability, we observed an overlap between 1W and 1 M samples, as well as between 1M and D samples. The fecal samples exhibited a clear separation between the hospitalization period and 3.5Y (Fig. 2B). Overall, the gut microbiota showed a discernible transformation from the 1W to the D samples. During the first few months of life, the composition of the microbiota of some infants exhibited a clustering with that of D samples, with the most pronounced shift occurring between 1M and D. Age accounted for 2.7% of the total variance in the compositional variation between these two time points, while it only accounted for 1.1% of the variance between 1W and 1 M samples, according to the weighted UniFrac distance (Table S2B).

### Gut microbiota community transitions

**Fig 3:**
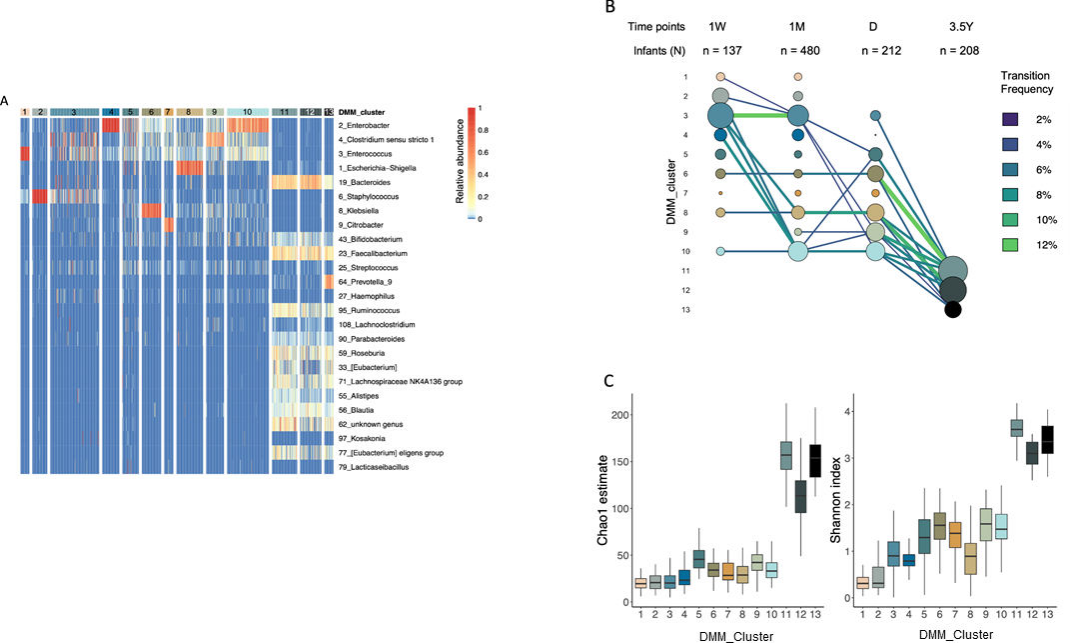
Development of the gut microbiota in very preterm infants, categorized by age-specific community types. (**A**) Heatmap showing the relative abundance of the 25 most dominant bacterial genera per Dirichlet multinomial mixture (DMM) cluster. Each DMM cluster was attributed a number (1 to 13) and was assigned a specific color that was consistently applied to the panels B and C. (**B**) Transition model showing the distribution of samples among the 13 identified DMM clusters for each sampling time point. The scale of circle sizes is determined by the frequency of each community type at each sampling point. Sampling points are 1 week (1W), 1 month (1M), NICU discharge (D), and 3.5 years of age (3.5Y). Edge size and color intensity are scaled by the transition frequency. Transitions with less than 2% frequency are not shown. (**C**) Box plots of alpha-diversity assessed by Chao1 and Shannon indexes for each DMM cluster. Box plots show the minimum and maximum values, 25% and 75% quartiles, and the median.

In order to characterize the evolution of the gut microbiota over time, we used a DDM clustering approach to identify the partitioning of the infants’ gut microbiota according to the sampling time point. The 1,037 fecal samples were divided into 13 DMM clusters (Fig. 3A). Clusters 1 and 2 were identified exclusively in 1W and 1 M samples (Fig. 3B) and were characterized by a high abundance of *Enterococcus* and *Staphylococcus*, respectively (Fig. 3A and B). Additionally, these clusters exhibited low alpha diversity (Fig. 3C). The majority of the 1 W samples belonged to cluster 3, which is characterized by a moderate abundance of *Clostridium sensu stricto 1, Enterococcus,* and *Staphylococcus,* rather than a dominance of a specific genus (Fig. 3A). The majority of samples from cluster 3 at 1W were classified in the same cluster at 1M, with a decrease in the prevalence over time. Cluster 4, which is characterized by the dominance of *Enterobacter*, predominantly grouped samples from 1W and 1M. Clusters 7 and 9, defined by the dominance of *Citrobacter* and *Clostridium sensu stricto 1*, respectively, included 1M and D samples as they were largely absent in 1 W samples. Clusters 6, 8, and 10 are characterized by a dominance of *Enterobacteriaceae* taxa, including *Klebsiella*, *Escherichia-Shigella*, and *Enterobacter*, respectively. Infants within these clusters exhibited a stable pattern without transitions and remained in the same cluster until D. At 3.5Y, samples were partitioned into three clusters, dominated by either both *Bacteroides* and *Faecalibacterium* (clusters 11 and 12) or *Prevotella_9* (cluster 13), with no discernible trends observed between clusters during the hospitalization period and at 3.5Y. We observed a trend of increased diversity (both Chao1 and Shannon) across the clusters with an overall difference across the clusters (*P* < 0.001). The clusters exhibiting the lowest levels of alpha-diversity (i.e., the less diverse) predominantly comprised 1 W samples, while those with the highest alpha-diversity (i.e., the most diverse) were predominantly composed of 3.5Y samples (Fig. 3C).

### Prediction of gut microbiota functional capabilities

**Fig 4:**
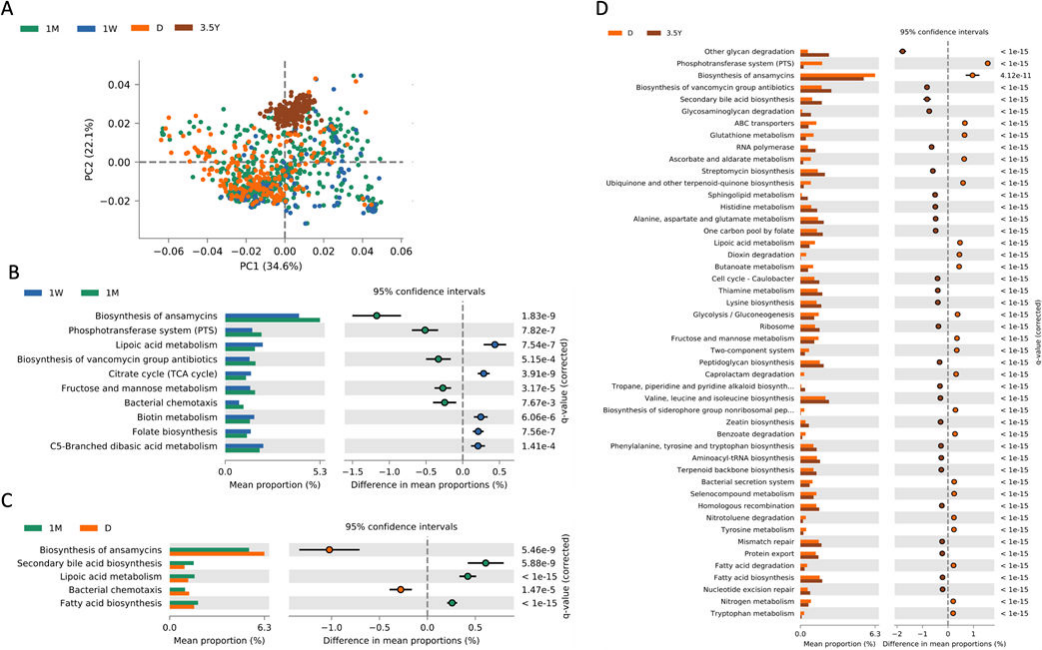
PICRUSt2 microbial metabolic functional pathway predictions across sampling time points. (**A**) PCA plot of the KEGG pathways. Dots are colored according to the sample time points at 1 week (1W; *n* = 137), 1 month (1M; *n* = 480), NICU discharge (D; *n* = 212), and 3.5 years of age (3.5Y; *n* = 208). (**B**) Extended bar plots represent the significantly different KEGG microbial metabolic functions between 1W and 1M. (**C**) Extended bar plots represent the significantly different KEGG microbial metabolic functions between 1M and D. (**D**) Extended bar plots represent the significantly different KEGG microbial metabolic functions between D and 3.5Y. Metabolic pathways are ranked according to the effect size. Only pathways with a *P*-value < 0.05 and a difference between proportions effect size > 0.2 are shown to capture highly affected functions. All inferences drawn from the functional pathway differences across sampling time points were evaluated using Welch’s test.

A PCA of the KEGG pathways was performed to observe the distribution pattern across the different sampling times. The PCA plot in Fig. 4A showed a similar clustering pattern to that of the NMDS plot of the taxonomic profile assessed by the beta-diversity (Fig. 2B). We observed a significant combined variance of PC1 and PC2 difference between the four time points (*P* < 0.001)**,** with the 1 W samples exhibiting the highest heterogeneity (Fig. 4A). A progressive spatial separation was observed between the 1W, 1M, and D samples, with the D samples clustering together (Fig. 4A). Between 1W and 1M, ten KEGG metabolic pathways were significantly differentially abundant (q < 0.001). At 1W, lipoic acid, biotin and C5-branched dibasic acid metabolism, citrate cycle, and folate biosynthesis were significantly overrepresented (q < 0.0001), while biosynthesis of ansamycins and vancomycin group antibiotics, phosphotransferase system (PTS) system, fructose and mannose metabolism, and bacterial chemotaxis were significantly underrepresented (q < 0.001) (Fig. 4B). Comparative analysis of the 1M and D samples showed that the ansamycin biosynthesis and bacterial chemotaxis pathways remained underrepresented at 1M (q < 0.0001), as did the secondary bile acid and fatty acid biosynthesis, and lipoic acid metabolism (q < 0.0001) (Fig. 4C). There was a clear separation between samples during the period of hospitalization and at 3.5Y (Fig. 4D). Between the D and 3.5Y samples, 48 metabolic pathways were significantly differentially abundant (q < 0.0001) (Fig. 4D). The most important difference was observed in the glycan degradation pathway, which was markedly more prevalent in the 3.5Y samples. Conversely, the biosynthesis of ansamycins, the PTS system, and the lipoic acid metabolism were less abundant compared to the D samples.

### Perinatal factors influencing gut microbiota

**Fig 5:**
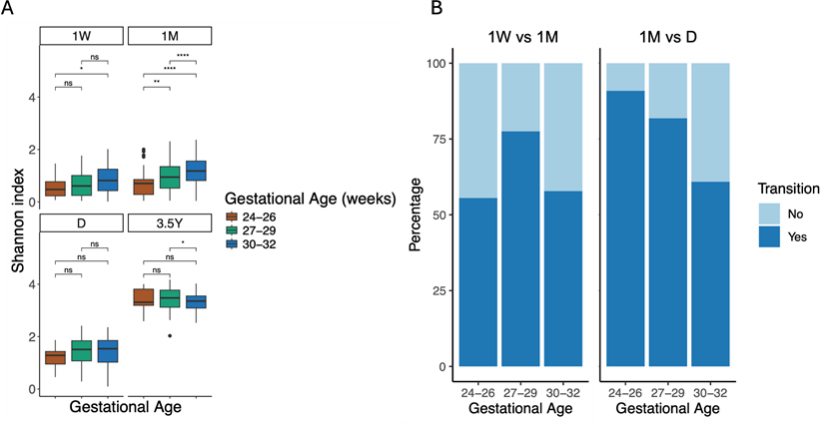
Effect of gestational age on the infant gut microbiota during the first 2 months of life. (**A**) Box plots of alpha-diversity assessed by the Shannon index according to gestational age groups (*P < 0.5; **P < 0.01; ***P < 0.001; ****P  <  0.0001; ns, nonsignificant; Kruskal–Wallis test). Only the 1 month (1M) sampling time point showed an overall difference in Shannon diversity between gestational age groups (*P* < 0.0001; Kruskal–Wallis test). The gestational age group colors are indicated in panel B. Box plots show minimum and maximum values, 25% and 75% quartiles, and the median. (**B**) Bar plots showing the rates of cluster transitions between 1 week (1W) and 1 month (1M) sampling time points (*n* = 113, *P* = 0.095) and between 1M and NICU discharge (D) time points (*n* = 158, *P* = 0.009) according to the gestational age categories. Numbers in each group category for each time point are available in Table S5B.

In order to investigate the influence of perinatal factors on the development of the very preterm infant gut microbiota, cross-sectional comparisons were performed using all sample time points (Table S3A). A significant effect of gestational age on the overall composition of the gut microbiota, as assessed by beta-diversity, was observed up to 1M (*P* = 0.001, both indexes). At 1M, infants with a higher gestational age exhibited higher richness and evenness (*P* < 0.01, both indexes) (Fig. 5; Table S3A). In particular, in infants born at the lowest gestational age (24–26 weeks), *Staphylococcus* remained the dominant genus at 1W and 1M, and *Bifidobacterium* was not detected up to D. Regarding the overall gut microbiota composition, birth weight was strongly associated with 1W (*P* = 0.001) and 1M (*P* = 0.001) (both indexes) (Table S4). A positive correlation was observed between birth weight and alpha diversity at 1M (*P* < 0.001, both indexes) (Table S3A). However, at D, infants born with higher weight exhibited lower richness (*P* = 0.024, Chao1 index), and no difference was observed in terms of evenness. The mode of delivery had no effect on alpha-diversity (Table S3A), but it was associated with the overall gut microbiota composition at 1W (*P* = 0.001), 1M (*P* = 0.011), and D (*P* = 0.045) (Bray–Curtis dissimilarity distance) (Table S4). The main effect was a reduction in the levels of *Bacteroides* throughout the hospital stay in the case of C-section delivery. This effect was transient and no longer observed at 3.5Y (Fig. S2). The administration of intrapartum antibiotics significantly impacted the composition of the gut microbiota at 1W (*P* = 0.049, Bray–Curtis dissimilarity distance) (Table S4). This was associated with a higher richness at 1W (*P* = 0.03, Chao 1 index) and a lower richness at 1M (*P* = 0.02, Shannon index) (Table S3A). Preconceptional maternal BMI was associated with the overall gut microbiota composition at 1M (*P* = 0.021) and 3.5Y (*P* = 0.003), and a similar trend was observed at 1W (Bray–Curtis dissimilarity distance) (Table S4). Infants born to obese mothers exhibited a higher diversity at 1M (*P* = 0.005) and D (*P* = 0.033) compared to those born to normal mothers (Table S3A). The same was observed for gut microbiota richness at 1W (*P* = 0.058). The pairwise PERMANOVA test showed that the observed difference was driven by neonates born to obese versus normal mothers at 1M (*P* = 0.036) and by neonates born to all categories versus normal mothers at 3.5Y (*P* = 0.017) (Table S4). Notably, the Bacteroidota phylum appeared to be more prevalent during the hospitalization period in infants born to underweight mothers (Fig. S3A). With regard to the Bacillota/Bacteroidota ratio (i.e., Firmicutes/Bacteroidetes ratio), infants born to underweight mothers exhibited a lower ratio due to a higher proportion of Bacteroidota during this period, particularly at 1M and D (Fig. S3B).

The practice of early skin-to-skin contact in the NICU was found to be associated with the overall gut microbiota composition at all the time points of the hospitalization sampling, according to either the Bray–Curtis or the weighted UniFrac distances (Table S4). According to alpha-diversity metrics, the gut microbiota of infants who benefited from the practice of early skin-to-skin contact showed higher richness and evenness at 1M (*P* = 0.001 and *P* = 0.002, respectively) (Table S3A). The main differences were observed in the proportions of *Staphylococcus* and *E. coli*. The former remained elevated at 1W and 1M, while the latter remained low at D in the absence of skin-to-skin contact (Fig. S4).

The impact of perinatal factors on the gut microbiota trajectory was investigated by assessing the transitions between two different DMM clusters between two consecutive sampling time points. The comparisons were made between the 1W and 1M groups and the 1M and D groups. As all infants transitioned between D and 3.5Y, the 3.5 Y samples were excluded from the analysis. The results indicated that neonates born at a lower gestational age were more likely to shift between two different clusters between 1M and D (*P* = 0.009), with 24–26, 27–29, and 30–32 GA groups showing a 90.9%, 81.8%, and 60.9% rates of transitions, respectively (Fig. 5B; Table S5B). A transition tendency was observed between 1W and 1M (*P* = 0.095) and in particular for the 27–29 GA group (Table S5A). Very preterm infants born by C-section were more likely to shift between 1W and 1M (*P* = 0.065) (Table S5A). With regard to the longitudinal associations between perinatal factors and alpha-diversity, only seven infants had complete series of fecal samples. Consequently, the analysis was limited to the 40 neonates with three sampling time points during the hospitalization period. It was observed that there were positive trends between microbiota diversity and gestational age (*P* = 0.076, Shannon index) and primary neonatal antibiotherapy (*P* = 0.094, Shannon index). A negative trend was observed for intrapartum antibiotic therapy (*P* = 0.064, Chao1 index) (Table S3B).

## DISCUSSION

The present study provides original data on the temporal dynamics and the potential functional activities of the gut microbiota of very preterm infants, during the hospitalization period and after NICU discharge. A progressive maturation of the gut microbiota over time was observed, characterized by an increase in alpha-diversity and a decrease in beta-diversity. This indicated a reduction in the variability of the gut microbiota. This was consistent with the observation that vey preterm infants transitioned between ten clusters during their hospitalization period to only three at 3.5 years of age, suggesting a stabilization of the gut microbiota. Furthermore, the analysis of the differentially abundant metabolic pathways reflected the differences observed in the composition and diversity of the gut microbiota between periods and was consistent with the gut microbiota shifting from a highly variable to a more stable adult-like gut microbiota.

The results of our study are consistent with those of previous studies that reported a dominance of Bacillota and Pseudomonadota in preterm infants, a low abundance of Bacteroidota during the hospitalization period, and a dominance of pathobionts (3, 15, 35–37). In the present cohort, infants also exhibited a markedly low abundance of *Bifidobacterium*. At the age of 3.5 years, there was a shift toward a greater abundance of Bacillota and Bacteroidota. Some studies have grouped gut microbiota bacterial patterns into clusters, each characterized by a dominant genus (20, 38). A subsequent analysis of all samples over time revealed that the gut microbiota communities underwent a gradual shift from ten to only three clusters by the time the infant reached 3.5 years of age. The ten clusters related to the hospitalization period were driven by *Enterobacter*, *Clostridium sensu stricto 1*, *Klebsiella*, *Escherichia*, *Enterococcus,* or *Staphylococcus* or both *Klebsiella* and *Enterococcus*. These genera are commonly found in the NICU environment, as previously reported in the literature (7, 16, 20, 38). Furthermore, the abundance of *Bifidobacterium* exhibited a gradual increase from week 1 until discharge from the NICU, in accordance with previous studies that have shown an association between postnatal age and *Bifidobacterium* colonization (7). However, no clear shift toward a *Bifidobacteriaceae*-dominated microbiota was observed. At the age of 3.5 years, the three identified clusters were dominated either by both *Bacteroides* and *Faecalibacterium* or by *Prevotella*. It has been suggested that preterm infants experience a delayed establishment of the gut microbiota, resulting in a reduction in the overall diversity during the first few months of life (17). Conversely, the bacterial diversity was found to increase following discharge from the NICU (39). Our data clearly demonstrate the transition of the microbiota from the early life and hospitalization period toward the establishment of gut microbiota communities that, at 3.5 years of age, are similar to those of adults.

In this study, we showed that perinatal factors influenced the composition of the gut microbiota. However, the observed associations depended on the sampling time or the considered period. The impact of *intrapartum* antibiotic administration was only observed at 1W. The impact of gestational age was more pronounced in extremely preterm infants, where potentially pathogenic bacteria, such as *Staphylococcus* and *Enterobacter*, were the dominant species at 1M. Furthermore, *Bifidobacterium*, which was present in low abundance at 1M, remained undetected until D for the most premature infants, as previously observed (40). As observed in full-term infants, C-section delivery was associated with a lower abundance of *Bacteroides*. Infants who did not benefit from the early practice of skin-to-skin contact exhibited an altered bacterial balance, with an increase in Staphylococci and a decrease in *E. coli.* It is unclear whether these changes in the gut microbiota constitute a dysbiosis that is associated with an increased risk of disease. A comparison of the preconceptional maternal BMI categories revealed that very preterm infants born from underweight mothers exhibited a lower Bacillota/Bacteroidota ratio compared to those born from normal and overweight/obese mothers. The Bacillota/Bacteroidota ratio is frequently positively associated with BMI, even in children (41–43). However, it is unclear whether the lower Bacillota/Bacteroidota ratio in infants born to underweight mothers is due to maternal BMI or other factors such as delivery mode. It has been observed that there is a lower likelihood of C-section deliveries among infants born to underweight mothers compared to those born to overweight or obese mothers. This may also contribute to the lower Bacillota/Bacteroidota ratio, and further research is therefore required.

No significant longitudinal associations were identified between perinatal factors and gut microbiota diversity. Nevertheless, trends were observed. This lack of significance may be attributed to insufficient statistical power, given the limited number of infants with a complete set of samples. However, our findings indicated that the preconceptional maternal BMI was the sole factor associated with the gut microbiota of infants during the hospitalization period and at 3.5 years of age. Our data indicate that the impact of the vertical transmission of an obesogenic gut microbiota may be noticeable as early as 1 month of life in a very preterm population. In addition, neonates born with a lower gestational age or delivered by C-section were more likely to transition between two different clusters during the hospitalization period due to the instability of their gut microbiota. Our results emphasize the importance of longitudinal cohorts and sampling time point when examining the impact of perinatal factors on the developing gut microbiota in preterm infants and the associated risk of disease.

In order to gain a more profound comprehension of the biological processes underlying the taxonomic associations, we conducted an analysis of the inferred potential functions of the microbial communities. The analysis revealed a temporal modification of the microbiota in terms of its functional capabilities. In particular, the biosynthesis of ansamycins and the bacterial chemotaxis pathways were found to be less abundant in the samples taken at 1 week compared to those taken at 1 month. Conversely, pathways involved in the biosynthesis of secondary bile acids and fatty acids, as well as the metabolism of lipoic acids, were more abundant. A similar pattern was observed between the samples taken at 1 month and those taken at NICU discharge. The biosynthesis of both ansamycin and vancomycin group antibiotics involves the incorporation of amino sugars derived from the fructose and mannose metabolism pathway (44). This pathway is linked to the PTS systems, which facilitate the transport and phosphorylation of sugars across the bacterial cell membrane (45). The PTS system also plays a role in sugar sensing and chemotaxis by regulating the expression of chemoreceptors and other chemotaxis proteins (45). The metabolism of branched-chain amino acids and the production of energy (ATP) are interconnected with the metabolism of lipoic acid, biotin, C5-branched dibasic acid, the citrate cycle, and folate biosynthesis (46). Moreover, lipoic acid metabolism is associated with the regulation of fatty acid synthesis. Both pathways are involved in regulating bile acid synthesis and metabolism (46). At 3.5 years, samples had 48 differentially abundant metabolic pathways in comparison to those at NICU discharge. The glycan degradation pathway represents a crucial and widespread process, whereby complex glycans are broken into simpler monosaccharides. This pathway is commonly used by the gut microbiota to degrade complex carbohydrates that cannot be digested by human enzymes alone (47). In contrast to the glycan degradation pathway, the biosynthesis of ansamycins, the PTS system, chemotaxis, lipoic acid metabolism, and fatty acids are relatively more specialized and less abundant pathways. The differential abundance of metabolic pathways observed in this study reflects the compositional and phylogenetic differences in the intestinal microbiota between periods. This is consistent with the gut microbiota undergoing a developmental shift from an immature to an adult-like state. Despite their differential abundance and specialization, this study highlighted the interconnections between these metabolic pathways and their contribution to overall metabolic homeostasis over time.

It should be noted that the study is subject to certain limitations. The utilization of the 16S rRNA gene sequencing approach limits this study to the description of gut bacterial composition and diversity. The use of shotgun metagenomics would have permitted a greater sequencing depth and the acquisition of information on microbiome functional profiles and species-level meaning. Although we did not have a complete set of samples for all infants, which impaired the statistical power of some analyses, we were still able to identify tendencies that require validation in future studies. The strengths of this study lie in its recruitment of a large, multicenter population of French infants born very preterm from a well-described nationwide birth cohort with late follow-up. This enabled the collection of valuable data and the drawing of meaningful conclusions.

In conclusion, the findings of this study reinforce the notion that the gut microbiota of very preterm infants exhibits distinctive characteristics. The study demonstrated the considerable variability of the gut microbiota in terms of its trajectory throughout the hospitalization period. Furthermore, the impact of perinatal factors on the transitions of the gut microbiota community and its potential functional activities over time is limited, with the exception of preconceptional maternal BMI. The degree of prematurity has been demonstrated to affect the instability of the gut microbiota, with lower gestational age preterm infants exhibiting greater instability. Nevertheless, following discharge from the NICU, all infants exhibit by the age of 3.5 years a microbiota community similar to that of adults.

## Data Availability

Personal data of children from the EPIFLORE cohort cannot be made publicly available for ethical reasons. They are available upon reasonable request from the authors under data-security conditions. The 16S rRNA gene reads are publicly available from the National Center for Biotechnology Information Sequence Read Archive (SRA) under Bioproject accession number PRJNA1146782.

## SUPPLEMENTAL MATERIAL

Supplemental material spectrum.01636-24-s0001.pdf Tables S1 to S5; Fig. S1 to S4.

ASM does not own the copyrights to Supplemental Material that may be linked to, or accessed through, an article. The authors have granted ASM a non-exclusive, world-wide license to publish the Supplemental Material files. Please contact the corresponding author directly for reuse.
