# Supplementary material for: Very preterm gut microbiota development from the first week of life to 3.5 years of age: a prospective longitudinal multicenter study
Source: Microbiol Spectr. 2025 Feb 19;13(4):e01636-24. doi: 10.1128/spectrum.01636-24 (PMC11960047; doi:10.1128/spectrum.01636-24)

Supplementary

Table S1. Differential abundance analysis between samples collected at 1 week (n=137), 1 month (n=480) and 3.5 years (n=208), and the NICU discharge time point (n=212).

otu.id	Genus	raw.pvalue	adj.pvalue	beta_1W	beta_1M	beta_3.5Y	se_1W	se_1M	se_3.5Y	W_1W	W_1M	W_3.5Y	raw.pvalue	adj.pvalue	effect_1M	effect_1W	effect_3.5Y	diff.btw_1M	diff.btw_1W	diff.btw_3.5Y	diff.win_1M	diff.win_1W	diff.win_3.5Y
Cluster_111	[Ruminococcus] torques group	8.62E-35	1.92E-34	-0.042	-0.030	1.586	0.035	0.028	0.129	-1.183	-1.066	12.278	2.85E-76	1.58E-75	-0.179	-0.310	1.191	-1.778	-0.984	5.681	5.336	5.052	4.312
Cluster_115	Subdoligranulum	1.19E-14	1.86E-14	-0.014	-0.171	0.930	0.089	0.054	0.148	-0.162	-3.186	6.301	1.22E-29	2.48E-29	-0.110	-0.255	0.801	-1.580	-0.648	4.582	5.333	5.327	5.040
Cluster_122	Corynebacterium	0	0	0.526	0.508	-2.630	0.142	0.079	0.052	3.702	6.433	-50.145	1.29E-33	3.05E-33	0.177	0.343	-0.724	2.110	1.153	-3.926	5.439	5.875	4.867
Cluster_126	Pantoea	0	0	-0.295	0.251	-3.085	0.112	0.094	0.078	-2.634	2.682	-39.703	1.65E-43	5.66E-43	-0.001	0.424	-0.865	2.549	-0.005	-4.515	5.507	5.455	4.793
Cluster_23	Faecalibacterium	0	0	-0.042	-0.384	3.906	0.135	0.084	0.103	-0.308	-4.569	37.920	1.14E-153	3.41E-152	-0.188	-0.576	2.266	-3.976	-1.318	8.268	6.276	6.479	3.482
Cluster_25	Streptococcus	7.09E-69	2.05E-68	-3.009	-2.315	-3.269	0.232	0.197	0.187	-12.974	-11.752	-17.524	1.77E-32	4.19E-32	-0.332	-0.183	-0.042	-1.257	-2.209	-0.242	6.340	6.039	4.781
Cluster_3	Enterococcus	0	0	-1.501	0.276	-7.910	0.284	0.180	0.146	-5.287	1.532	-54.103	3.14E-212	3.04E-210	-0.041	0.622	-2.173	5.320	-0.369	-12.657	8.321	8.572	5.584
Cluster_35	Bifidobacterium	1.70E-49	4.15E-49	-2.207	-1.622	0.090	0.253	0.246	0.256	-8.715	-6.588	0.353	8.97E-36	2.44E-35	-0.329	-0.376	0.753	-2.896	-2.238	4.886	7.186	6.316	5.424
Cluster_42	Lachnoclostridium	7.98E-97	2.49E-96	-0.696	-0.472	1.315	0.165	0.157	0.163	-4.210	-3.012	8.049	5.67E-65	2.71E-64	-0.292	-0.372	1.352	-2.437	-1.803	5.397	5.623	5.403	3.643
Cluster_56	Blautia	0	**0**	-0.324	-0.288	2.584	0.100	0.095	0.111	-3.238	-3.040	23.277	2.13E-122	3.19E-121	-0.249	-0.450	2.093	-2.853	-1.494	6.868	5.806	5.440	3.187
Cluster_57	[Ruminococcus] gnavus group	1.08E-12	1.66E-12	-0.195	-0.120	0.888	0.083	0.082	0.158	-2.353	-1.464	5.623	3.48E-48	1.35E-47	-0.176	-0.246	0.913	-1.443	-0.988	4.736	5.242	4.975	4.759
Cluster_59	Roseburia	3.62E-162	1.41E-161	-0.026	-0.098	2.836	0.075	0.056	0.115	-0.343	-1.764	24.604	1.72E-126	2.79E-125	-0.185	-0.399	1.760	-2.559	-1.171	7.221	5.922	5.598	3.889
Cluster_6	Staphylococcus	0	0	3.145	1.770	-5.213	0.305	0.178	0.143	10.318	9.945	-36.552	7.67E-188	3.07E-186	0.614	0.590	-1.892	5.144	5.340	-10.845	8.145	8.345	5.647
Cluster_64	Prevotella_9	8.73E-05	0.00011355	0.230	-0.278	-0.641	0.160	0.094	0.236	1.440	-2.969	-2.713	0.00423452	0.0044142	0.102	-0.070	-0.007	-0.442	0.697	-0.055	5.797	5.940	6.938
Cluster_98	Lachnospira	1.17E-28	2.27E-28	-0.030	-0.011	1.505	0.043	0.036	0.133	-0.704	-0.291	11.302	5.24E-65	2.46E-64	-0.151	-0.297	1.059	-1.651	-0.909	5.429	5.307	5.058	4.532

Fecal sampling time points: 1W=1 week; 1M= 1 month; D= NICU discharge; 3.5Y= 3.5 years.
All comparisons are made against the reference being NICU discharge (D) fecal sampling point.
ANCOM-BC: beta= coefficient obtained from the ANCOM-BC log linear (natural log) model (log-transformed change in abundance), se = standard error of the beta, W = test statistic (beta/se).
ALDEx2: effect= per-feature effect size, diff.btw= per-feature median difference between the two conditions, diff.win= per-feature maximum median difference between Dirichlet instances within conditions.
adj.pvalue = Global p value corresponding to Chi-square test for ANCOM-BC and glm ANOVA for ALDEx2 with False discovery rate (FDR) correction.

Table S2. Alpha (A) and (B) beta diversities between age groups within the full cohort.

(A) Alpha diversity

	N	Chao1	Shannon	Test
1W vs 1M	*137 (1W), 480 (1M)*	**0.0013**	**6.30E-08**	Wilcoxon rank-sum
1W vs D	*137 (1W), 212 (D)*	**< 2e-16**	**< 2e-16**	Wilcoxon rank-sum
1W vs 3.5Y	*137 (1W), 208 (3.5Y)*	**< 2e-16**	**< 2e-16**	Wilcoxon rank-sum
1M vs D	*480 (1M), 212 (D)*	**< 2e-16**	**8.80E-15**	Wilcoxon rank-sum
1M vs 3.5Y	*480 (1M), 208 (3.5Y)*	**< 2e-16**	**< 2e-16**	Wilcoxon rank-sum
D vs 3.5Y	*212 (D), 208 (3.5Y)*	**< 2e-16**	**< 2e-16**	Wilcoxon rank-sum

	N	Chao1	Shannon	Test
1W vs 1M	*113*	**0.0003**	**1.84E-10**	Wilcoxon signed-rank
1W vs D	*51*	**8.20E-05**	**9.40E-09**	Wilcoxon signed-rank
1W vs 3.5Y	*39*	**7.28E-12**	**3.64E-12**	Wilcoxon signed-rank
1M vs D	*158*	**1.99E-08**	**2.01E-10**	Wilcoxon signed-rank
1M vs 3.5Y	*141*	**< 2.2e-16**	**< 2.2e-16**	Wilcoxon signed-rank
D vs 3.5Y	*57*	**5.28E-11**	**5.28E-11**	Wilcoxon signed-rank

(B) Beta diversity

Bray-Curtis

N=	*137*	*480*	*212*	*208*
	1W	1M	D	3.5Y
1W	NA	**0.001**	**0.001**	**0.001**
1M	0.015	NA	**0.001**	**0.001**
D	0.049	0.017	NA	**0.001**
3.5Y	0.165	0.148	0.201	NA

P-values in the upper triangle and R2 values in the lower triangle for each comparison

Wunifrac

N=	*137*	*480*	*212*	*208*
	1W	1M	D	3.5Y
1W	NA	**0.008**	**0.001**	**0.001**
1M	**0.011**	NA	**0.001**	**0.001**
D	0.075	0.027	NA	**0.001**
3.5Y	0.478	0.445	0.631	NA

P-values in the upper triangle and R2 values in the lower triangle for each comparison

Fecal sampling time points: 1W: 1 week, n = 137; 1M: 1 month, n = 480; D: NICU discharge, n = 212; 3.5Y: 3.5 years, n = 208.

Table S3. Cross-sectional (A) and longitudinal (B) associations between perinatal factors and alpha diversity, as measured by the Chao1 and Shannon indexes

(A) Cross-sectional association

**Gestational age**	N	Chao1				Shannon			
Wilcoxon rank-sum test		P-value	Median			P-value	Median		
OTU level			**24-26**	**27-29**	**30-32**		**24-26**	**27-29**	**30-32**
1W	136	0.202	17.170	25.430	23.170	0.057	0.471	0.606	0.818
1M	479	**0.000**	19.250	23.500	31.000	**0.000**	0.702	0.946	1.180
D	211	0.527	39.830	39.430	38.000	0.187	1.284	1.510	1.536
3.5Y	207	0.112	148.500	142.500	137.300	0.065	3.313	3.476	3.353

**Delivery mode**	N	Chao1			Shannon		
Wilcoxon rank-sum test		P-value	Median		P-value	Median	
			**Vaginal**	**Cesarean**		**Vaginal**	**Cesarean**
1W	135	0.754	23.330	23.000	0.798	0.690	0.721
1M	478	0.978	26.500	26.000	0.677	1.038	1.051
D	211	0.602	40.000	38.000	0.103	1.261	1.562
3.5Y	206	0.711	140.600	139.560	0.211	3.440	3.349

**Birth weight**	N	Chao1		Shannon	
Spearman correlation		P-value	r	P-value	r
1W	137	0.679	0.036	0.616	0.043
1M	480	**0.000**	0.300	**0.000**	0.271
D	212	**0.024**	-0.155	0.779	-0.019
3.5Y	208	0.946	0.005	0.415	-0.057

**Primary antibiotherapy**	N	Chao1			Shannon		
Wilcoxon rank-sum test		P-value	Median		P-value	Median	
			**No**	**Yes**		**No**	**Yes**
1W	114	0.634	25.090	23.330	0.342	0.874	0.649
1M	423	0.430	28.000	26.000	0.635	1.061	1.010
D	195	0.574	39.250	38.250	0.085	1.590	1.349
3.5Y	187	0.175	136.750	142.100	**0.025**	3.310	3.433

**Mother antenatal antibiotherapy**	N	Chao1			Shannon		
Wilcoxon rank-sum test		P-value	Median		P-value	Median	
			**No**	**Yes**		**No**	**Yes**
1W	134	0.331	21.250	24.250	0.415	0.769	0.619
1M	476	0.969	26.100	26.000	0.137	1.073	1.012
D	207	0.174	41.000	36.620	0.176	1.592	1.332
3.5Y	204	0.122	137.850	145.600	0.138	3.323	3.450

**Mother intra partum antibiotherapy**	N	Chao1			Shannon		
Wilcoxon rank-sum test		P-value	Median		P-value	Median	
			**No**	**Yes**		**No**	**Yes**
1W	120	**0.031**	21.000	25.920	0.945	0.719	0.657
1M	419	0.276	28.000	25.000	**0.025**	1.091	0.909
D	194	0.287	37.000	38.500	0.372	1.562	1.388
3.5Y	179	0.278	138.200	145.800	0.776	3.364	3.424

**Skin to skin practice**	N	Chao1			Shannon		
Wilcoxon rank-sum test		P-value	Median		P-value	Median	
			**No**	**Yes**		**No**	**Yes**
1W	132	0.561	21.000	23.330	0.719	0.721	0.747
1M	453	**0.001**	23.200	28.460	**0.002**	0.937	1.110
D	201	0.090	42.000	36.120	0.250	1.476	1.506
3.5Y	198	0.351	142.400	138.300	0.104	3.438	3.337

**Human milk consumption**	N	Chao1		Shannon	
Wilcoxon rank-sum test		P-value	**Median**	**P-value**	**Median**

			No	Yes		No	Yes
1W	90	0.487	25.670	21.000	0.429	0.504	0.721
1M	299	0.161	22.620	26.380	0.884	1.048	1.082
D	137	0.666	36.750	38.000	0.480	1.542	1.514

**Preconceptional maternal BMI**	N		Chao1					Shannon			
Kruskal-Wallis test		P-value	Median				P-value	Median			
			**Normal**	**Underweight**	**Overweight**	**Obese**		**Normal**	**Underweight**	**Overweight**	**Obese**
1W	127	0.058	20.000	17.000	27.080	24.750	0.231	0.671	0.774	0.734	1.071
1M	447	0.368	26.000	25.500	28.170	30.250	**0.005**	0.953	1.270	1.090	1.346
D	195	0.596	39.430	30.880	35.330	37.500	**0.033**	1.476	1.205	1.407	1.635
3.5Y	195	0.093	140.300	119.330	142.600	149.500	0.181	3.367	3.248	3.498	3.505

Preconceptional maternal BMI for significant Kruskal-Wallis test

Dunn's test		Shannon	Z	P-value FDR corrected
1M	307	N_vs_Un	-2.080	**0.038**
	359	N_vs_Ov	-2.130	**0.050**
	336	N_vs_Ob	-2.844	**0.013**
	141	Ov_vs_Ob	0.839	0.301
	112	Ov_vs_Un	-0.611	0.325
	89	Ob_vs_Un	0.064	0.475
D	120	N_vs_Un	1.492	0.081
	151	N_vs_Ov	-0.510	0.305
	145	N_vs_Ob	-2.321	**0.030**
	76	Ov_vs_Ob	1.559	0.089
	51	Ov_vs_Un	0.661	0.097
	45	Ob_vs_Un	2.634	**0.025**

Fecal sampling time points: 1W: 1 week; 1M: 1 month; D: NICU discharge; 3.5Y: 3.5 years.
BMI: body mass index; N: Normal; Un: Underweight; Ov: Overweight; Ob: Obese
P-value ≤ 0.05 are marked in bold.

(B) Longitudinal associations

	Chao1			Shannon		
	**Beta***	**95% CI**	**p-value**	**Beta***	**95% CI**	**p-value**
**Gestational age (categories in weeks) (38)**			0.637			0.076
24-26	—	—		—	—	
27-29	4.0	-11, 19		0.21	-0.39, 0.81	
30-32	6.4	-7.9, 21		0.51	-0.06, 1.1	
**Delivery mode (38)**			0.332			0.415
Vaginal	—	—		—	—	
Cesarean	-4.1	-12, 4.2		0.15	-0.21, 0.51	
**Birth weight (g) (38)**	0.01	-0.01, 0.02	0.323	0.00	-0.01, 0.02	0.150
**Sex (38)**			0.659			0.387
Boys	—	—		—	—	
Girls	1.6	-5.7, 9.0		0.14	-0.17, 0.44	
**Neonatal primary antibiotherapy (35)**			0.742			0.094
No	—	—		—	—	
Yes	1.3	-6.2, 8.8		-0.27	-0.59, 0.05	
**Mother antenatal antibiotherapy (36)**			0.188			0.949
No	—	—		—	—	
Yes	4.6	-2.2, 11		-0.01	-0.29, 0.27	
**Mother intra partum antibiotherapy (33)**			0.062			0.263
No	—	—		—	—	
Yes	7.4	-0.38, 15		-0.19	-0.52, 0.14	
**Skin-to-skin practice (36)**			0.587			0.754
Yes	—	—		—	—	
No	-2.6	-12, 6.7		-0.06	-0.46, 0.33	
**Human milk consumption (28)**			0.341			0.420
No	—	—		—	—	
Yes	4.9	-5.2, 15		-0.17	-0.57, 0.24	
**Preconceptional maternel BMI (35)**			0.531			0.145
Underweight	-11	-36, 15		-0.03	-1.1, 1.0	
Normal	—	—		—	—	
Overweight	2.7	-7.2, 13		-0.35	-0.75, 0.06	
Obese	-6.1	-17, 4.5		0.26	-0.17, 0.69	

Mixed linear regression model.
Complete case longitudinal analysis performed on infant with time points available at 1W, 1M and D.
(N) = number of subjects in each model; CI = Confidence Interval; BMI = Body Mass Index
*Beta estimates adjusted for mother age, maternal education, and country of birth of the mother.

Table S4. PERMANOVA results on the impact of perinatal factors on the gut microbiota within different age group.

**Gestational age**	N	R2		P-value FDR corrected	
		Bray-Curtis	Wunifrac	Bray-Curtis	Wunifrac
1W	136	0.032	0.104	**0.001**	**0.001**
1M	479	0.020	0.066	**0.001**	**0.001**
D	211	0.009	0.011	0.496	0.299
3.5Y	207	0.011	0.015	0.265	0.091

**Delivery mode**		R2		P-value FDR corrected	
		Bray-Curtis	Wunifrac	Bray-Curtis	Wunifrac
1W	135	0.025	0.060	**0.001**	**0.001**
1M	478	0.005	0.005	**0.011**	0.086
D	211	0.008	0.009	**0.045**	0.143
3.5Y	206	0.006	0.004	0.218	0.534

**Birth weight**		R2		P-value FDR corrected	
		Bray-Curtis	Wunifrac	Bray-Curtis	Wunifrac
1W	137	0.031	0.076	**0.001**	**0.001**
1M	480	0.016	0.072	**0.001**	**0.001**
D	212	0.006	0.005	0.277	0.290
3.5Y	208	0.006	0.008	0.170	0.091

**Neonatal primary antibiotherapy**		R2		P-value FDR corrected	
		Bray-Curtis	Wunifrac	Bray-Curtis	Wunifrac
1W	114	0.011	0.016	0.239	0.151
1M	423	0.003	0.002	0.167	0.501
D	195	0.007	0.007	0.115	0.213
3.5Y	187	0.007	0.006	0.155	0.333

**Mother antenatal antibiotherapy**		R2		P-value FDR corrected	
		Bray-Curtis	Wunifrac	Bray-Curtis	Wunifrac
1W	134	0.005	0.001	0.83	0.977
1M	476	0.003	0.001	0.182	0.829
D	207	0.008	0.008	0.085	0.158
3.5Y	204	0.005	0.008	0.455	0.133

**Mother intra partum antibiotherapy**		R2		P-value FDR corrected	
		Bray-Curtis	Wunifrac	Bray-Curtis	Wunifrac
1W	120	0.014	0.005	**0.049**	0.496
1M	419	0.003	0.003	0.126	0.236
D	194	0.006	0.008	0.235	0.164
3.5Y	179	0.007	0.010	0.141	0.086

Skin to skin practice		R2		P-value FDR corrected	
		Bray-Curtis	Wunifrac	Bray-Curtis	Wunifrac
1W	132	0.012	0.003	0.054	0.701
1M	453	0.005	0.011	**0.027**	**0.013**
D	201	0.010	0.007	**0.032**	0.219
3.5Y	198	0.005	0.009	0.592	0.099

Human milk consumption		R2		P-value FDR corrected	
		Bray-Curtis	Wunifrac	Bray-Curtis	Wunifrac
1W	90	0.008	0.008	0.743	0.471
1M	299	0.003	0.003	0.463	0.334
D	137	0.017	0.009	**0.018**	0.302

Preconceptional maternal BMI		R2		P-value FDR corrected	
		Bray-Curtis	Wunifrac	Bray-Curtis	Wunifrac
1W	127	0.031	0.011	0.083	0.813
1M	447	0.010	0.005	**0.021**	0.643
D	195	0.014	0.027	0.525	0.069
3.5Y	195	0.024	0.164	**0.003**	0.259

Preconceptional maternal BMI		Pairwise adonis	R2 Bray-Curtis	P-value FDR corrected
1M	307	N_vs_Un	0.013	0.092
	359	N_vs_Ov	0.011	0.061
	336	N_vs_Ob	0.013	**0.036**
	141	Ov_vs_Ob	0.021	0.477
	112	Ov_vs_Un	0.024	0.622
	89	Ob_vs_Un	0.038	0.269
3.5Y	153	N_vs_Un	0.028	**0.009**
	171	N_vs_Ov	0.025	**0.017**
	156	N_vs_Ob	0.030	**0.003**
	43	Ov_vs_Ob	0.077	0.307
	40	Ov_vs_Un	0.085	0.228
	25	Ob_vs_Un	0.132	0.350

Fecal sampling time points: 1W: 1 week; 1M: 1 month; D: NICU discharge; 3.5Y: 3.5 years.
BMI = Body Mass Index; N: Normal; Un: Underweight; Ov: Overweight; Ob: Obese
P-value ≤ 0.05 are marked in bold.

Table S5. Transition data between DMM clusters and fecal sampling time points (A) and the specificity for gestational age (B)

(A) Fisher's exact test

	1W vs 1M		1M vs D	
	N	P-value	N	P-value
Delivery mode	113	0.065	158	1.000
Gestational age	113	0.095	158	**0.009**
Neonatal primary antibiotherapy	93	0.109	146	0.401
Mother antenatal antibiotherapy	111	0.227	155	0.725
Mother intra partum antibiotherapy	100	0.170	146	1.000
Skin to skin practice	108	1.000	150	0.345
Mother BMI before pregnancy	108	0.554	149	0.392
Human milk consumption*	72	0.244	88	0.812

Fecal sampling time points: 1W, 1 week; 1M, 1 month; D, NICU discharge.
Neonatal primary antibiotherapy: first 78h of life
*Human milk consumption was dichotomized as children receiving human milk at time point t and t+1 and those, not receiving human milk at t+1.

(B) Frequencies of transitions per categories of gestational age

**Gestational age (categories in weeks)**	**24-26**	**27-29**	**30-32**
	1W - 1M (N=113)		
	**(N=9)**	**(N=40)**	**(N=64)**
**Transition**			
No	4 (44.4%)	9 (22.5%)	27 (42.2%)
Yes	5 (55.6%)	31 (77.5%)	37 (57.8%)
	1M - D (N=158)		
	**(N=11)**	**(N=55)**	**(N=92)**
**Transition**			
No	1 (9.1%)	10 (18.2%)	36 (39.1%)
Yes	10 (90.9%)	45 (81.8%)	56 (60.9%)

Number of transitions according to the gestational age category.
Fecal sampling time points: 1W, 1 week; 1M, 1 month; D, NICU discharge.

Figure S1. Gut microbiota composition of very preterm infants from 1 week of life to 3.5 years of age according to gestational age. Relative abundance of taxa at the level of the 20 most abundant genera, represented by their phylum affiliation**.**

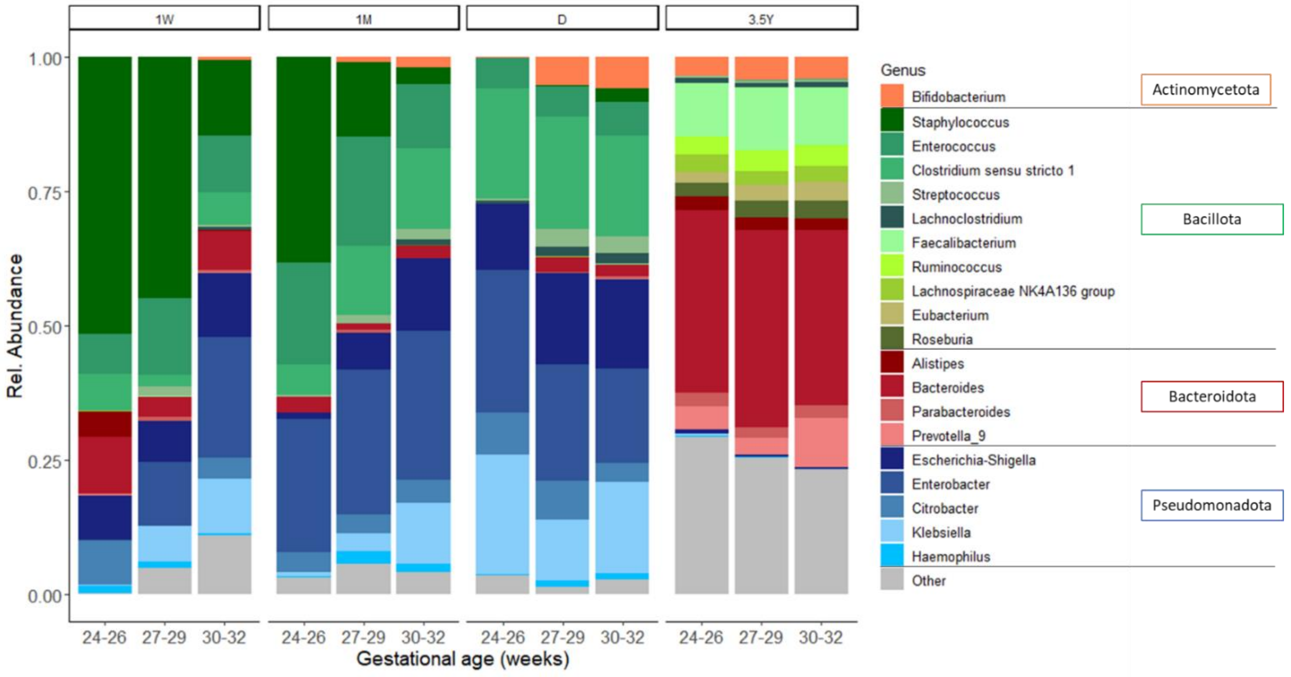

Figure S2. Gut microbiota composition of very preterm infants from 1 week of life to 3.5 years of age according to vaginal (V) or cesarean (C) delivery mode. Relative abundance of taxa at the level of the 20 most abundant genera, represented by their phylum affiliation**.**

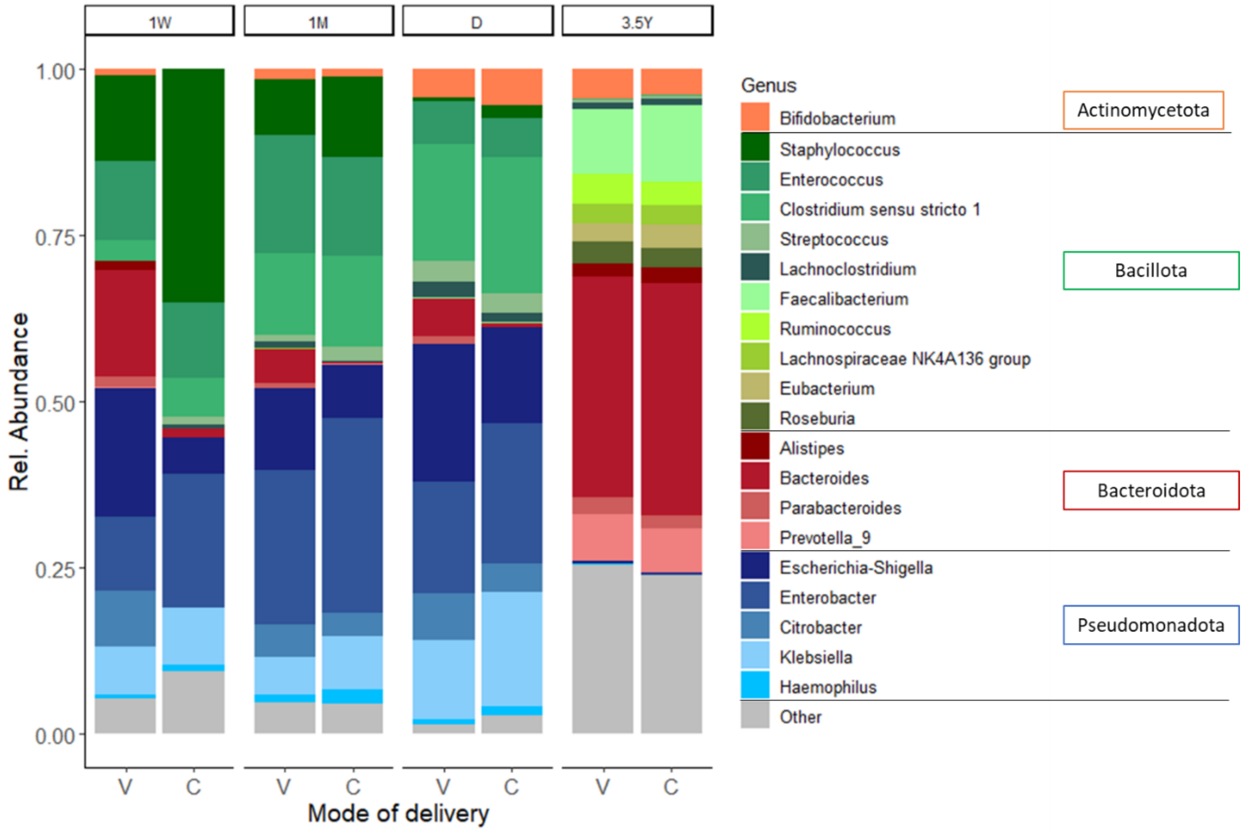

Figure S3. (A) Gut microbiota composition at the phylum level of the very preterm infants from 1 week of life to 3.5 years of age according to preconceptional maternal BMI categories. (B) Bacillota/Bacteroidota ratio among different preconceptional maternal BMI categories during the hospitalization period.

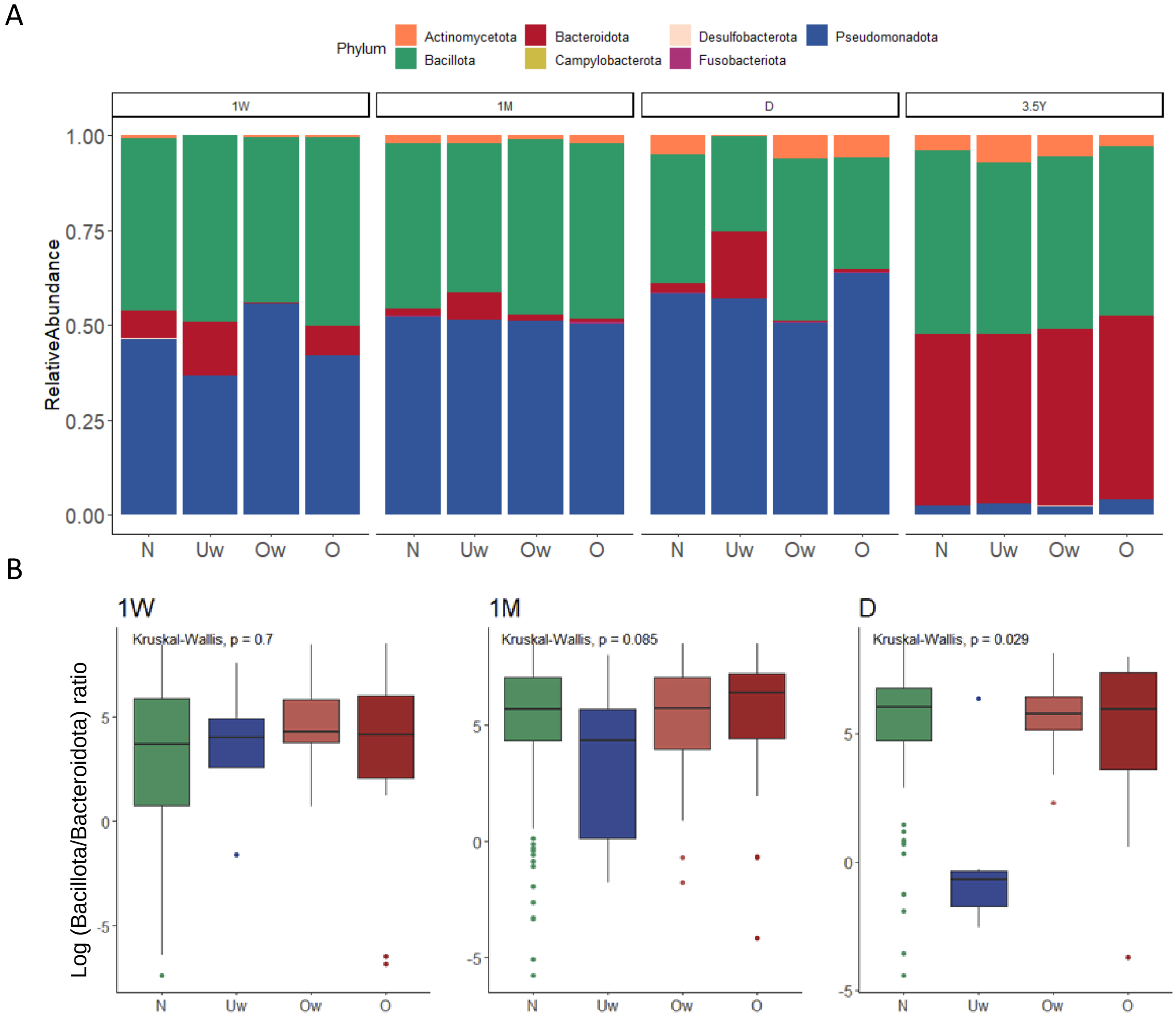

Bacillota/Bacteroidota ratio is expressed as log ratio of Bacillota abundance divided by that of Bacteroidota. Differences were tested using the Kruskall-Wallis test comparison among the different Preconceptional maternal BMI categories.

BMI = Body Mass Index, N = normal, Uw = underweight, Ow = overweight, O = obese.

Figure S4. Gut microbiota composition of very preterm infants from 1 week of life to 3.5 years of age according to early skin-to-skin contact. Relative abundance of taxa at the level of the 20 most abundant genera, represented by their phylum affiliation**.**

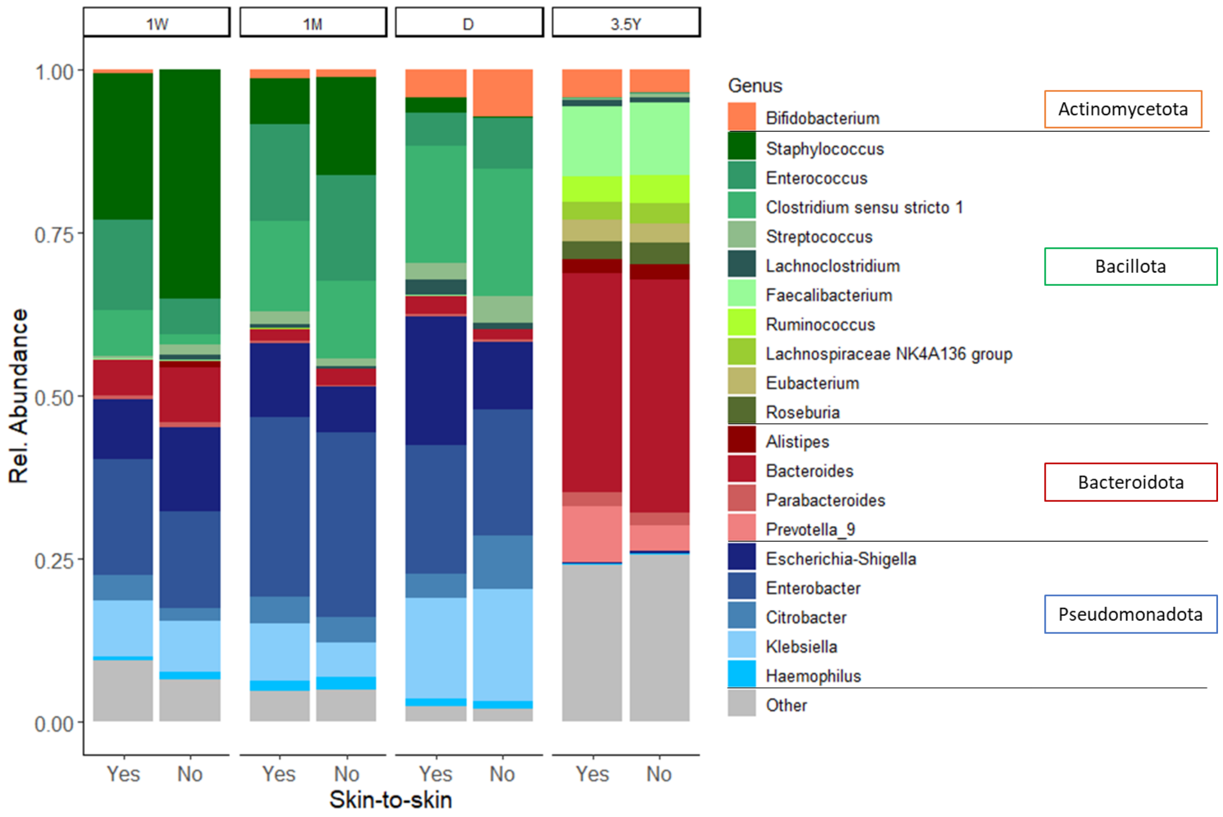

Supplement: Supplemental material — Tables S1 to S5; Fig. S1 to S4. [file spectrum.01636-24-s0001.pdf]
